# Biomimetics as a Functional Engineering Framework for Mechanical Systems: A PRISMA-Guided Systematic Mapping of Sensing, Inspection, Access Robotics, and Condition Monitoring (2016–2026)

**DOI:** 10.3390/biomimetics11050346

**Published:** 2026-05-15

**Authors:** Cristóbal Galleguillos Ketterer, Nicolás Norambuena Ortega, José Luis Valín

**Affiliations:** Escuela de Ingeniería Mecánica, Facultad de Ingeniería, Pontificia Universidad Católica de Valparaíso, Avenida Brasil 2950, Valparaíso 2340025, Chile; nicolas.norambuena@pucv.cl (N.N.O.); jose.valin@pucv.cl (J.L.V.)

**Keywords:** biomimetics, mechanical systems, inspection robotics, condition monitoring, tribology, sensing, systematic mapping, PRISMA 2020

## Abstract

Mechanical engineering systems must sense, inspect, and navigate constrained environments and operate adaptively under uncertainty—requirements that map structurally onto capabilities achieved by biological systems through distributed sensing, morphology-driven locomotion, multimodal perception, and decentralised control. Biomimetics can therefore be interpreted not merely as a source of design inspiration but also as a functional engineering framework relevant to industrial monitoring, inspection, maintenance, and autonomous operation. This study presents a PRISMA 2020-guided systematic mapping review of the biomimetics literature explicitly relevant to mechanical-engineering functions over the decade 2016–2026. A Scopus corpus of 11,114 records was screened through a two-stage abstract-level process. After deduplication and broad relevance filtering, a stricter eligibility audit retained 505 studies assignable to five predefined functional clusters: robotics and access (235 records; 46.5%), mechanical surfaces and tribology (141; 27.9%), sensing and monitoring (106; 21.0%), vision and inspection (14; 2.8%), and control and computation (9; 1.8%). Publication output accelerated markedly after 2022, with 2025 yielding the highest annual count. The principal gap identified is not a shortage of biomimetic concepts, but their limited consolidation into deployable industrial inspection and maintenance architectures. A translational taxonomy connecting biological principles, engineering abstractions, enabling technologies, and mechanical use cases is proposed as an interpretive structuring tool for future research prioritisation and technology-readiness discussion.

## 1. Introduction

Mechanical engineering systems underpin critical industrial infrastructure—pipelines, rotating machinery, turbines, pressure vessels, and structural assets—whose reliable operation depends on continuous sensing, periodic inspection, and timely maintenance decisions. As these assets grow more complex and their operating environments become more demanding, conventional engineering approaches face persistent limitations: accessing confined geometries, detecting incipient faults under noise and vibration, and enabling autonomous decision-making without continuous human intervention [1,2].

Biological systems have addressed equivalent functional challenges over evolutionary timescales. Bats localise targets at centimetre-level resolution through acoustic pulse propagation and echo analysis. Fish lateral-line mechanoreceptors resolve hydrodynamic disturbances at sub-Pascal pressure levels [3,4]. Geckos and insects access vertical and inverted surfaces through dry adhesion mechanisms with load capacities orders of magnitude beyond their body weight [5]. Snakes and earthworms navigate irregular confined passages through undulatory and peristaltic locomotion that passively adapts to geometric constraints [6]. Arthropods integrate distributed tactile and chemical sensing for environmental mapping [7]. In each case, the biological solution provides a validated existence proof that a demanding engineering function—detection, access, locomotion, sensing, control—can be achieved with limited energy, mass, and computation.

Biomimetics, the deliberate translation of biological principles into engineered systems, has expanded substantially as a research field over the past decade [8,9]. Existing reviews have addressed specific sub-domains in depth: bioinspired flow and hair-cell sensors [4,10], dry-adhesion materials and climbing robotics [5,6], adaptive control strategies for legged platforms [11], and nature-inspired sensing materials [7]. Broader bibliometric analyses confirm rapid growth in publication output and diversification across engineering subdisciplines [12] but do not distinguish mechanical-engineering functions from biomedical, materials, or computational contributions. No prior mapping study has applied a mechanically grounded functional taxonomy to characterise which operational requirements—sensing, inspection, access, and maintenance—are served by the biomimetics literature as a whole, nor identified the structural gaps that separate current research from deployable industrial systems. The present study addresses this gap. However, the published literature is highly heterogeneous: a broad Scopus query anchored in biomimetic terminology retrieves contributions from biomedical engineering, tissue science, drug delivery, advanced materials, robotics, and computation alongside the mechanical-engineering applications that practitioners in industrial sensing, inspection, and maintenance require.

The central thesis of this study is that biomimetics, when applied to mechanical engineering, functions most productively not as a design inspiration catalogue but as a functional engineering framework for systems that must perceive condition, inspect complex assets, navigate constrained environments, adapt control strategies, and support maintenance and autonomous decision-making. This reinterpretation positions biomimetic research within the operational demands of Industry 4.0-oriented maintenance [13], structural health monitoring [2], non-destructive testing (NDT), and inspection robotics [6], rather than treating it as a peripheral design philosophy.

To examine this proposition, the present study conducts a PRISMA 2020-guided systematic mapping review of Scopus-indexed biomimetics literature explicitly relevant to mechanical-engineering functions over the period 2016–2026. The review is designed as an abstract-level evidence map rather than as a full systematic review with study-level effect synthesis or formal quality appraisal. Its objectives are (i) to characterise the temporal evolution, document-type composition, and source-journal distribution of the mechanically relevant biomimetics corpus; (ii) to identify the dominant functional clusters within that corpus using a predefined mechanical-function framework; (iii) to develop a translational taxonomy linking biological principles, engineering abstractions, enabling technologies, and representative industrial applications; and (iv) to identify research gaps that constrain the translation of biomimetic concepts into deployable sensing, inspection, access, and maintenance systems. The study therefore advances biomimetics as an interpretive functional framework supported by the mapped structure of the literature, rather than as a conclusively demonstrated unified doctrine of engineering practice.

## 2. Materials and Methods

### 2.1. Review Design

This study is a PRISMA 2020-guided systematic mapping review [14,15]. Its purpose is to map the thematic and functional structure of a research field rather than to synthesise study-level intervention effects, compare outcome estimates, or perform formal risk-of-bias appraisal. Given the heterogeneity of biomimetic research across system types, experimental scales, and application contexts, a mapping design is methodologically more defensible than a conventional effectiveness review or meta-analysis. The review was conducted at the abstract-metadata level using bibliographic records exported from Scopus; no full-text retrieval was performed. The review was not prospectively registered, which is acknowledged as a limitation.

### 2.2. Eligibility Criteria

Inclusion and exclusion criteria were defined a priori and are reported in Table 1. Records were included when they presented biomimetic, bioinspired, bio-inspired, bionic, or nature-inspired framing and demonstrated relevance to at least one mechanical-engineering function: sensing, acoustic perception, machine vision, chemical detection, inspection, fault diagnosis, structural or condition monitoring, locomotion, navigation, access to confined environments, or adaptive control. Records were excluded when the primary domain was biomedical, clinical, tissue engineering, drug delivery, or chemistry without plausible translation to mechanical systems.

### 2.3. Information Sources and Search Strategy

The information source was Scopus (Elsevier), queried in April 2026. The search strategy was designed for broad identification of biomimetic literature with possible mechanical-engineering relevance, rather than for formally benchmarked recall and precision optimisation against an external gold-standard set. The resulting corpus was subsequently refined through a two-stage abstract-level screening pipeline. Scopus was used as the sole database to preserve procedural consistency across bibliographic metadata fields; however, this choice likely underrepresents literature indexed preferentially in Web of Science, IEEE Xplore, and Compendex, especially conference-led engineering contributions. The full Scopus query used for corpus identification is reproduced below exactly as executed:


TITLE-ABS-KEY ( ( "biomimetics" OR "bioinspired" OR "bio-inspired"
 OR "bionic" OR "nature-inspired" OR "biomimicry"
 OR "biologically inspired" )
AND ( "mechanical engineering" OR "inspection" OR "monitoring"
 OR "robotics" OR "locomotion" OR "condition monitoring"
 OR "fault diagnosis" OR "maintenance" OR "vibration"
 OR "acoustics" OR "machine vision" OR "tribology"
 OR "pipeline" OR "turbine" OR "bearing" OR "structural health"
 OR "ndt" OR "nondestructive" OR "climbing robot"
 OR "snake robot" OR "soft robot" ) )
AND PUBYEAR > 2015 AND PUBYEAR < 2027
AND DOCTYPE ( ar OR re OR cp )


This query returned 11,114 records. Export fields included title, abstract, author keywords, index keywords, source title, document type, DOI, EID, publication year, and author affiliation. Bibliographic screening, deduplication, descriptive counting, and figure generation were performed using Python 3.11 with pandas 2.2, NumPy 1.26, Matplotlib 3.8, NetworkX 3.2, and scikit-learn 1.4. Scopus (Elsevier B.V., Amsterdam, The Netherlands; https://www.scopus.com, accessed on 6 April 2026) was the bibliographic database used for the export. No additional databases were searched; this single-database limitation is acknowledged in Section 4.5.

### 2.4. Selection Process

Selection proceeded through two machine-assisted abstract-level stages operating on title, abstract, and keyword metadata. Stage 1 functioned as a broad relevance screen intended to retain records with explicit biomimetic framing and plausible linkage to mechanical-engineering functions. After deduplication, this stage reduced the Scopus corpus to a narrower candidate set by excluding records lacking sufficient mechanical-functional relevance at the metadata level. Stage 2 applied stricter eligibility rules to remove records whose dominant contribution remained biomedical, chemical, tissue-engineering, energy-storage, photocatalytic, or otherwise non-mechanical despite biomimetic terminology. Borderline cases were adjudicated by author consensus using the predefined mechanical-functional scope of the review. No formal inter-rater agreement statistic was computed; this is acknowledged as a methodological limitation.

The included corpus was re-audited against the predefined five-cluster analytical framework. Records that could not be assigned robustly to one of the five functional clusters with sufficient scope fidelity were removed from the final mapped dataset rather than retained in a residual category. The final corpus therefore contains 505 studies assigned to five predefined functional clusters. The study identification and screening process is summarised in the revised PRISMA-style flow diagram; because screening was conducted at the abstract-metadata level, no full-text reports were retrieved. The completed PRISMA 2020 checklist is provided in Supplementary Table S1.

### 2.5. Data Collection and Topic Assignment

Each record was assigned to one of five a priori functional clusters (Table 2) by rule-based keyword matching followed by manual adjudication of ambiguous cases. For records whose titles, abstracts, or keywords indicated more than one plausible function, assignment was made to the dominant primary engineering function explicitly emphasised in the record metadata. Each record received one primary cluster assignment.

### 2.6. Synthesis Approach

Synthesis was descriptive and visual: frequency counts, temporal distributions, source-journal rankings, keyword co-occurrence analysis, and cluster sizes were computed and displayed graphically. No quantitative meta-analysis was performed. The synthesis objective was to map the structure of the field and identify underserved functional areas.

### 2.7. Protocol and Registration

This review was not prospectively registered. PROSPERO registration was not applicable because the present study is outside the registry scope, which is limited to systematic reviews addressing human health and wellbeing outcomes. Therefore, retrospective registration in PROSPERO was not appropriate.

## 3. Results

### 3.1. Search and Selection Results

**Figure 1:**
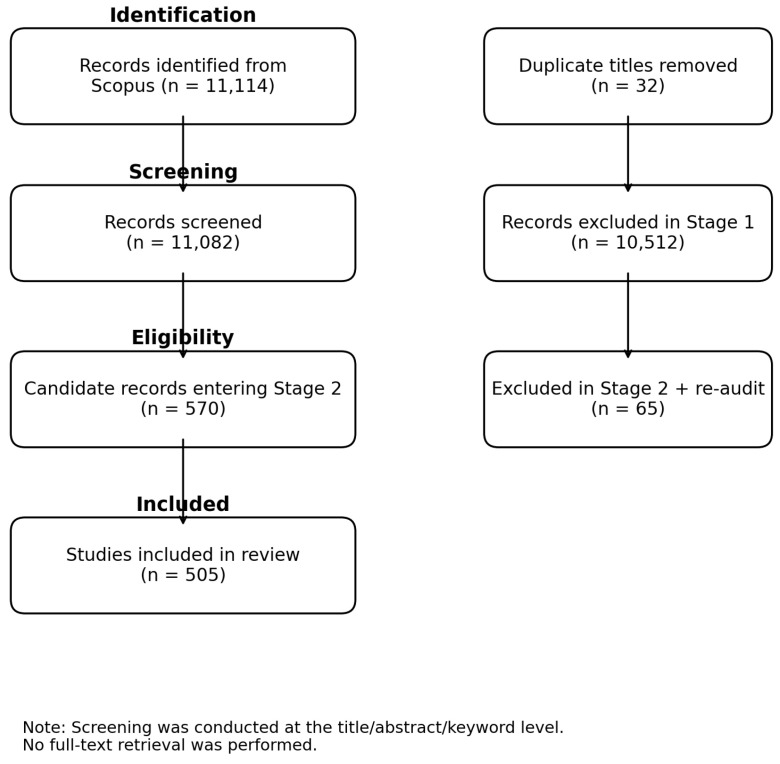
PRISMA 2020-style flow diagram for the two-stage machine-assisted screening pipeline. Both stages operated on title, abstract, and keyword fields; no full-text retrieval was performed. This deviation from the standard PRISMA 2020 flow is acknowledged explicitly as a methodological limitation.

The Scopus export contained 11,114 records. After removal of 32 duplicate titles, 11,082 records remained for screening. The broad mechanical-industrial relevance filter (Stage 1) retained 570 records (5.1% of the deduplicated corpus). The strict eligibility screen (Stage 2) retained 505 records (88.6% of the Stage 1 set; 4.7% of the deduplicated corpus). The PRISMA-style flow is presented in Figure 1.

### 3.2. Temporal Distribution

**Figure 2:**
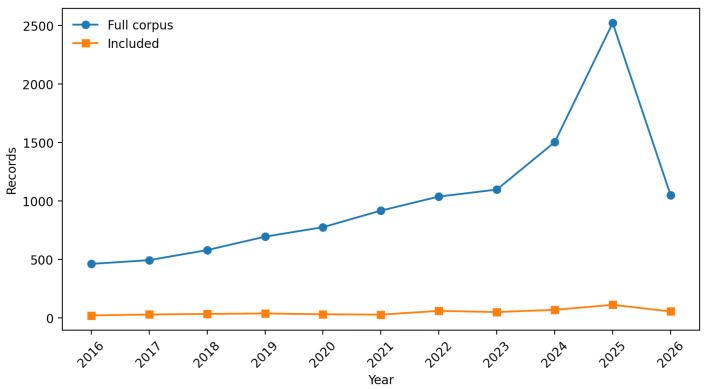
Annual publication counts for the full Scopus corpus (11,114 records) and the final included set (505 records), 2016–2026. The 2026 count is partial (search conducted in April 2026).

Publication output in the included set increased progressively over the study period (Figure 2). Annual counts were relatively stable between 2016 and 2021 (range: 19–36 records per year), then increased sharply from 2022 onwards. The year 2025 yielded the highest count (110 records), with 2026 already at 53 records with the year not yet complete at the time of the search. This post-2022 acceleration is consistent with the convergence of advanced manufacturing, soft robotics, machine-learning-based sensing, and the broader diffusion of bioinspired design practice into mainstream engineering research.

### 3.3. Document Types and Source Journals

Within the included set, articles dominate (323 records; 64.0%), followed by conference papers (103; 20.4%) and reviews (79; 15.6%) (Table 3). The relatively high review proportion (15.6%) compared with the full corpus reflects the maturity of certain biomimetic subfields—particularly bioinspired dry adhesion, tribology, and in-pipe inspection robotics—where synthesis articles are now appearing alongside primary research.

**Figure 3:**
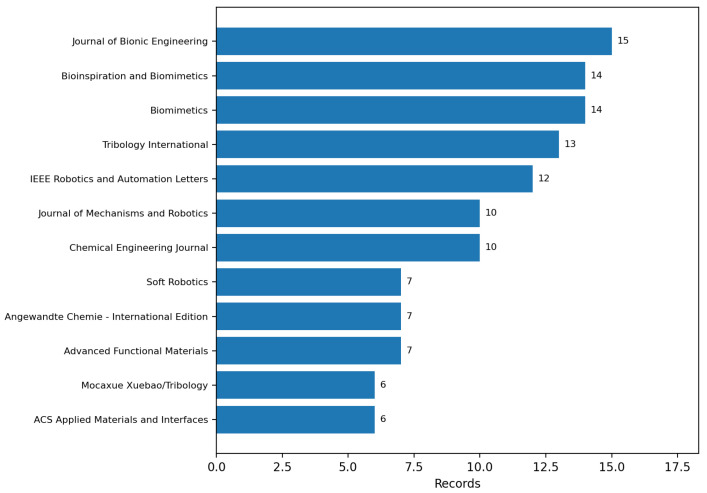
Top source journals in the included set, regenerated directly from the final included dataset. The co-presence of biomimetics-focused and engineering-specialised venues reflects the translational character of the literature and the partial segregation of publication communities.

The top source journals are listed in Table 4 and visualised in Figure 3. The ranking was regenerated directly from the final included dataset to ensure one-to-one consistency between the tabulated counts and the visualisation. *Journal of Bionic Engineering* leads (15 records), followed by *Bioinspiration and Biomimetics* and *Biomimetics* (14 each), and *Tribology International* (13). The co-presence of biomimetics-focused journals and engineering- specialised venues indicates that the mapped literature remains distributed across partially segregated publication communities.

### 3.4. Keyword Landscape

**Figure 4:**
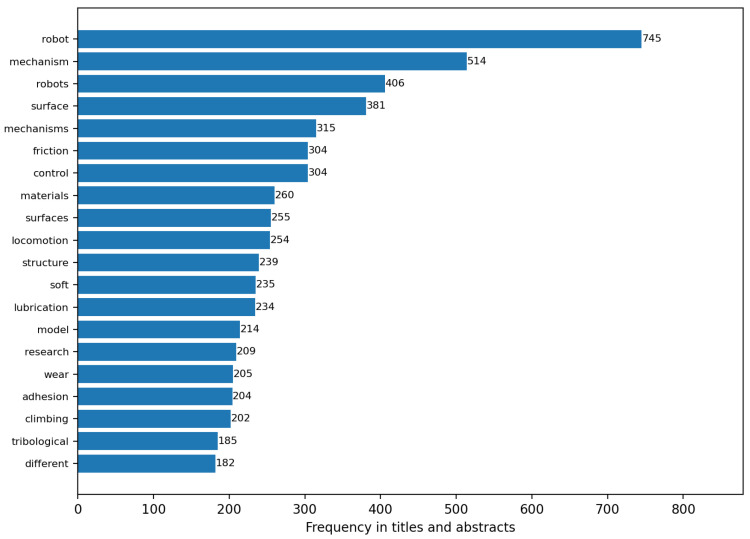
Top 20 terms in titles and abstracts of the included set, ranked by frequency. Robotics, tribology, and adhesion vocabulary dominate; maintenance, condition monitoring, and fault diagnosis terms are present but comparatively infrequent.

**Figure 5:**
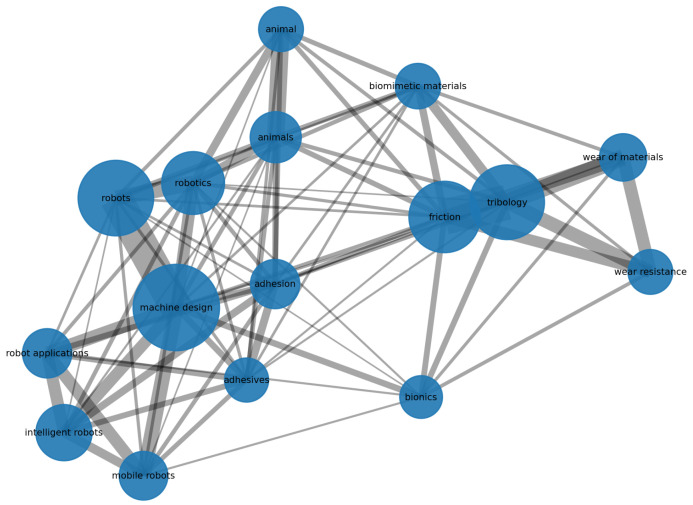
Keyword co-occurrence network for the included set. Node size is proportional to keyword frequency; edge weight is proportional to co-occurrence count. The robotics-locomotion cluster is more internally dense than the sensing-monitoring cluster, and cross-cluster connectivity between sensing and robotics is sparse, indicating limited integration of these function domains in the current literature.

The most frequent terms in titles and abstracts of the included set (Figure 4) centre on friction, biomimetic, bio-inspired design, legged robots, climbing robots, tribology, soft robotics, and adhesion. Terms directly associated with industrial condition monitoring—fault diagnosis, structural health monitoring, predictive maintenance, and nondestructive testing—are present but occupy the lower frequency tier. The keyword co-occurrence network (Figure 5) shows limited cross-connectivity between the robotics and sensing clusters, indicating that sensing and locomotion functions are not being co-designed in most included studies.

### 3.5. Functional Cluster Distribution

**Figure 6:**
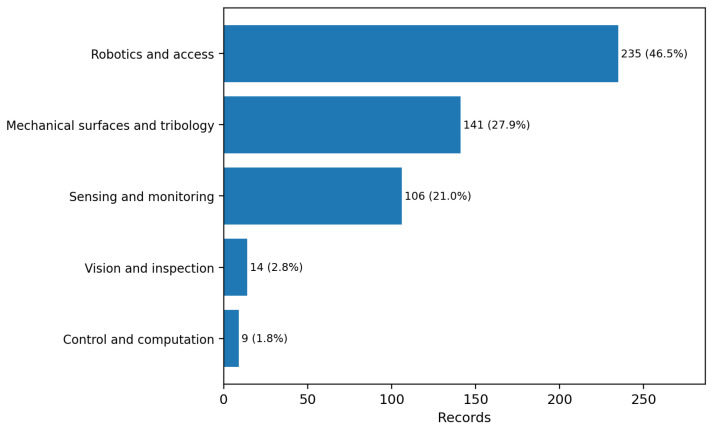
Functional cluster distribution of the included set (n=505). Robotics and access constitutes the plurality (46.5%); vision and inspection is the smallest named cluster (2.8%) despite being most directly relevant to automated non-destructive testing and asset inspection.

Table 5 and Figure 6 present the distribution of the 505 included records across the five predefined functional clusters. Robotics and access is the largest cluster (235 records; 46.5%), followed by mechanical surfaces and tribology (141; 27.9%) and sensing and monitoring (106; 21.0%). Vision and inspection (14; 2.8%) and control and computation (9; 1.8%) remain markedly underrepresented despite their relevance to industrial monitoring, non-destructive evaluation, and autonomous inspection workflows.

### 3.6. Document Type Distribution

**Figure 7:**
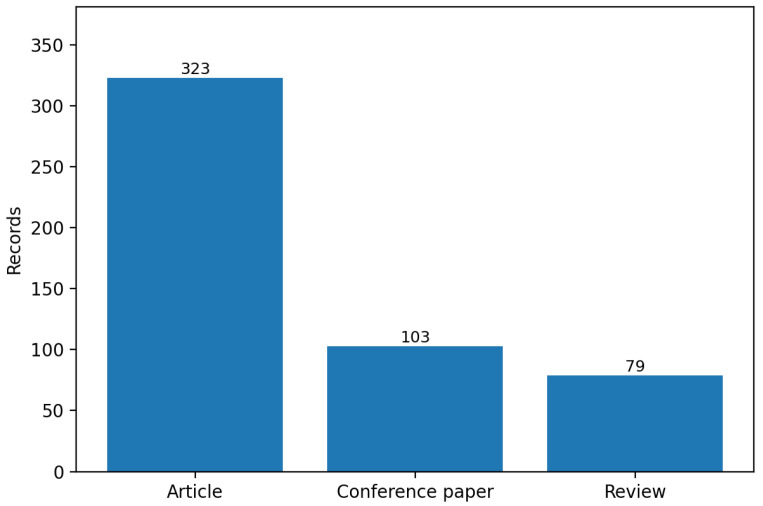
Document type distribution in the included set (n=505). Articles and conference papers collectively account for 84.4% of included records; the elevated review proportion (15.6%) reflects maturing synthesis activity in bioinspired tribology and inspection robotics.

Figure 7 shows the document type distribution across the full included set. The high proportion of review articles (15.6%) relative to the full Scopus corpus reflects active synthesis effort in bioinspired tribology, adhesion, and in-pipe inspection robotics.

### 3.7. Translational Taxonomy

Table 6 presents the translational taxonomy constructed from the included evidence, connecting biological principles to engineering abstractions, enabling technologies, representative industrial use cases, and indicative technology readiness levels (TRL). TRL estimates are based on the authoring team’s synthesis of the included literature and should be interpreted as approximate relative rankings rather than certified assessments.

**Figure 8:**
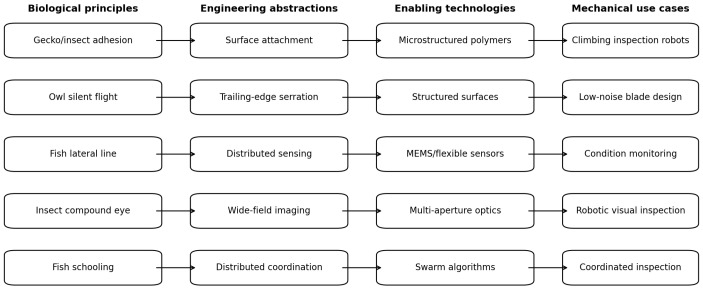
Conceptual structure of the translational taxonomy (this figure). Biological principles (**left**) are mapped through engineering abstractions and enabling technologies to mechanical-engineering use cases (**right**). The layout groups pathways consistently with the five functional clusters in Table 2.

Figure 8 illustrates the conceptual structure of the proposed taxonomy and has been redrawn to improve structural readability in grayscale viewing.

## 4. Discussion

### 4.1. Biomimetics as a Functional Engineering Framework

The central interpretive argument of this review is that biomimetics, when read through the lens of mechanical-engineering functions, can be productively organised as a functional framework for systems that must perceive, navigate, inspect, and adapt under industrial constraints. The evidence mapped here supports this framing as a useful analytical synthesis of the literature rather than as a conclusively demonstrated unified property of the field. The five functional clusters correspond to major operational requirements in condition monitoring, inspection, access, and maintenance; however, their uneven distribution also shows that the field remains fragmented and only partially integrated at the level of deployable engineering systems.

The functional alignment is not superficial. Bat echolocation and ultrasonic NDT share the physical substrate of acoustic pulse propagation and echo analysis; the difference lies in the transduction substrate and signal-processing chain, not in the underlying detection logic [10]. Lateral-line mechanoreception and distributed pressure sensor arrays for pipeline flow monitoring share the requirement for spatial resolution of small pressure differentials; the translation barrier is primarily miniaturisation and manufacturing, not concept validity [3]. Compound insect vision and wide-field machine vision share the aperture–resolution trade-off managed through multi-element optical design [19]. In each case, the biological system provides a validated existence proof that the functional requirement can be met with limited energy, mass, and computational overhead—constraints equally relevant in industrial deployments.

### 4.2. Functional Cluster Analysis

#### 4.2.1. Robotics and Access

The robotics-and-access cluster (235 records; 46.5%) is the largest and most internally diverse. It spans gecko- and insect-inspired dry-adhesion climbing robots for vertical-surface inspection [5], earthworm-inspired peristaltic robots for in-pipe traversal [6,17], snake-like robots for duct and confined-space access, cephalopod-inspired soft underwater vehicles [18], and insect-scale crawling robots for narrow-gap penetration [20,21]. The mechanical rationale for biological inspiration is strong: legged, crawling, and undulatory locomotion offer stability on irregular surfaces, passive compliance under geometric constraints, and lower actuator demands than wheeled or tracked alternatives.

The principal limitation of this cluster, as observed consistently across included papers, is the gap between demonstrated locomotion capability and deployed inspection functionality. Most papers report locomotion performance metrics (maximum slope angle, adhesion force, payload capacity, and swimming speed) rather than inspection outcomes (defect detection rate, coverage efficiency, false-alarm rate, communication reliability under industrial electromagnetic interference) [22,23,24]. This locomotion-first orientation leaves the integration with sensing payloads, data transmission, and maintenance decision workflows largely unaddressed [25,26]. The priority future research direction for this cluster is the co-design of locomotion morphology and sensing payload as a unified inspection system, with performance metrics defined by operational inspection outcomes rather than locomotion benchmarks alone.

#### 4.2.2. Mechanical Surfaces and Tribology

The surfaces-and-tribology cluster (141 records; 27.9%) encompasses bioinspired adhesion, friction, wear resistance, anti-icing, and aeroacoustic performance. The owl-inspired trailing-edge serration literature is the most industrially mature sub-theme, with multiple included studies reporting aeroacoustic performance in wind tunnel conditions applicable to wind turbine and fan blade design [27]. Bioinspired dry-adhesion surfaces for climbing robots [5,16] partially overlap with the robotics-and-access cluster, pointing to a productive path where surface function and locomotion function are co-designed as an integrated system.

Tribology-focused papers in this cluster predominantly address friction reduction, lubrication, and wear at machine interfaces, inspired by biological lubrication mechanisms (insect joints, synovial analogues) [28,29,30,31]. This is an industrially immediate application with clear maintenance relevance—reducing bearing and gear wear directly extends asset service life—yet explicit linkage to condition monitoring or maintenance scheduling frameworks is absent from most included papers. Future work in this cluster should prioritise embedding bioinspired surface solutions within condition-monitoring architectures, closing the loop between wear-state sensing and maintenance decision support.

#### 4.2.3. Sensing and Monitoring

The sensing-and-monitoring cluster (106 records; 21.0%) is the most directly relevant to the industrial application goals articulated in the introduction. It encompasses bioinspired transduction principles applied to structural condition monitoring, vibration analysis, acoustic emission, tactile sensing, and chemical detection. Key sub-themes include piezoelectric sensors inspired by hair-cell mechanoreception for rotating-machinery fault characterisation [32], flexible strain sensors for motion and structural monitoring [33,34], butterfly-wing-inspired photonic sensors for structural colour change under load [35,36], and biomimetic acoustic emission sensing for early-stage crack detection [37].

The translation path from the bioinspired sensing concept to maintenance-relevant measurement is better documented in this cluster than in robotics-and-access. Several included papers report performance against application-relevant benchmarks (sensitivity, noise floor, bandwidth, cross-sensitivity). However, field validation under real industrial electromagnetic interference, temperature cycling, and contamination is rarely reported, and explicit integration with maintenance decision-support systems is not observed in any included paper. The priority future direction is the translation of laboratory-validated bioinspired sensors into field-deployable architectures integrated with SHM data pipelines and predictive maintenance workflows.

#### 4.2.4. Vision and Inspection

Vision-and-inspection is the smallest named cluster (14 records; 2.8%), a finding that is arguably the most operationally significant result of this review. Visual inspection remains the most widely practised NDT method in mechanical engineering, and bioinspired imaging strategies—compound-eye optics, foveated cameras, event-driven vision, polarisation-sensitive imaging—offer genuine advantages for robotic inspection platforms in terms of weight, power, and field of view [19,38]. The small size of this cluster should not be interpreted uncritically as proof of technological scarcity. It likely reflects a combination of genuine underrepresentation within the mapped mechanical corpus and terminology fragmentation across optics, photonics, computational imaging, and inspection engineering literatures. Bioinspired vision papers are often published in venues that do not align with mechanical-engineering indexing conventions, while inspection robotic papers may use functionally relevant imaging hardware without biomimetic framing in titles, abstracts, or keywords. The priority future direction is dedicated systematic mapping of bioinspired imaging contributions across optics, photonics, and inspection-engineering venues, combined with cross-disciplinary benchmarking against standard NDT detection tasks.

#### 4.2.5. Control and Computation

The control-and-computation cluster (nine records; 1.8%) is the smallest overall, which merits careful interpretation. Bioinspired control algorithms (ant colony optimisation, particle swarm, genetic algorithms, neuromorphic control) are present throughout the included literature but appear within papers classified under the robotics, sensing, or surfaces clusters. As a standalone function directed at mechanical-system control, this cluster is underrepresented—only papers where bioinspired computation is the primary contribution, applied to a clearly mechanical control problem, received this assignment. The low count reflects the need for dedicated integration work at the interface of bioinspired control and industrial mechanical systems, rather than an absence of relevant computational techniques [11,39]. The priority future direction is the development of bioinspired adaptive controllers explicitly validated on industrial mechanical platforms—bearing monitoring, in-pipe navigation, and structural inspection—with performance assessed against conventional control baselines.

### 4.3. Priority Research Gaps

Five priority gaps were identified from systematic patterns across the included literature.

Gap 1. Weak integration of sensing payload with access locomotion.

Robotics papers report locomotion metrics; sensing papers report sensor performance. Papers that co-design and co-validate locomotion and sensing for a defined inspection task are rare. Industrial inspection systems require both simultaneously: a climbing robot that cannot reliably transmit acoustic or optical data has no operational value. The research opportunity is multi-functional platform design where locomotion morphology and sensing strategy are treated as a coupled design problem, with performance metrics defined by inspection requirements—detection rate, coverage efficiency, data transmission reliability—rather than locomotion benchmarks alone.

Gap 2. Absence of field-deployment validation.

The overwhelming majority of included papers report laboratory or prototype-scale results. Field testing under industrial conditions (electromagnetic interference, temperature extremes, contamination, and vibration from adjacent machinery) is not reported in any included paper. Technology readiness levels for most included platforms appear to be TRL 3–5; the path to TRL 7–8 (prototype validated in operational environment) is not addressed. Structured deployment studies in representative industrial environments—refineries, offshore platforms, pipelines, and rotating machinery halls—reporting not only performance but also failure modes, maintenance requirements, and operator interface effectiveness would constitute high-impact contributions.

Gap 3. Underrepresentation of condition monitoring and maintenance decision support.

Explicit condition monitoring, fault diagnosis, and predictive maintenance applications constitute only 21.0% of the included evidence, and within this cluster, field-deployable architectures that close the loop from sensing to maintenance action are not observed. The integration of bioinspired sensing with SHM data management frameworks [2] and with maintenance decision-support systems represents the frontier that the current literature has not yet systematically addressed.

Gap 4. Fragmentation across publication venues.

The keyword co-occurrence network (Figure 5) shows sparse cross-connectivity between robotics, sensing, and tribology clusters. Source journals are similarly segregated: biomimetics journals, robotics journals, tribology journals, and NDT journals do not systematically cross-cite. Practitioners in industrial inspection and maintenance are unlikely to encounter relevant biomimetic research published in optics or materials venues. Review articles explicitly targeting inspection and maintenance practitioners—structured around operational requirements rather than biological organism families—would reduce this fragmentation and accelerate technology transfer.

Gap 5. Absence of standardised benchmarking.

Performance metrics across included papers are heterogeneous: climbing robot studies report maximum slope angle and adhesion force; sensing papers report sensitivity and bandwidth; and vision papers report accuracy on image datasets. No common industrial inspection benchmark (detection rate, false-alarm rate, access geometry, area coverage rate, operating time) is used across the included literature. Without common benchmarks, inter-study comparison is impossible and field progress cannot be rigorously assessed. The development of standardised inspection and monitoring benchmarks for bioinspired platforms—analogous to manipulation benchmarks in service robotics—is a pre-competitive research priority.

### 4.4. Connections to Intelligent Maintenance and Industry 4.0

The functional framework proposed in this review is directly compatible with the trajectory of Industry 4.0 and intelligent maintenance [13]. Condition-based and predictive maintenance depend on continuous, reliable, and interpretable sensing data from assets that are frequently in difficult-to-access locations. Biomimetics contributes to this trajectory at three levels. At the sensor level, bioinspired transduction offers advantages in sensitivity, energy efficiency, spatial resolution, and multimodality relevant to continuous industrial monitoring [32,35]. At the platform level, bioinspired locomotion enables inspection access to environments inaccessible to conventional sensors [5,6]. At the control level, bioinspired adaptive architectures can manage the uncertainty, noise, and environmental variability that characterise industrial inspection environments [11]. The integration of these three levels within a unified maintenance workflow is the frontier that the current 505 included studies have not yet systematically reached.

### 4.5. Limitations

Several methodological limitations constrain the confidence that can be placed in the present results.

First, the review used a single database (Scopus). Web of Science, IEEE Xplore, Compendex, and Google Scholar may contain relevant records not captured, particularly IEEE-indexed conference papers. The single-database design likely introduces an undercount in engineering-conference literature indexed by IEEE but not Scopus.

Second, both screening stages operated at the abstract level; no full-text retrieval was performed. Records whose relevance to mechanical engineering is evident only from full-text content may have been incorrectly excluded. Conversely, records with mechanically suggestive abstracts but non-mechanical core contributions may have been incorrectly included.

Third, topic assignment to functional clusters was performed by one primary reviewer with team adjudication; no inter-rater reliability statistic was computed. The five-cluster taxonomy involves judgement calls at cluster boundaries.

Fourth, no structured post hoc analysis of the excluded-record population was performed; therefore, the disciplinary and document-type structure of exclusion bias cannot be quantified directly from the present review.

Fifth, the review was not prospectively registered.

Sixth, the TRL estimates in Table 6 are indicative, based on the authors’ synthesis of the included literature, not on a formal TRL assessment protocol.

These limitations do not invalidate the evidence map but should be considered when interpreting the gap analysis and the translational taxonomy.

## 5. Conclusions

This study maps the structure of biomimetics research relevant to mechanical engineering functions across 2016–2026, based on a PRISMA 2020-guided two-stage screening of 11,114 Scopus records yielding 505 included studies. The principal findings are as follows.

1. Publication output in the included domain accelerated markedly from 2022 onwards, with 2025 yielding the highest annual count (110 records), indicating an accelerating research front in bioinspired mechanical engineering.
2. The functional cluster distribution is dominated by robotics and access (46.5%) and mechanical surfaces and tribology (27.9%), with sensing and monitoring (21.0%) representing the cluster most directly relevant to condition monitoring. Vision and inspection (2.8%) and control and computation (1.8%) are markedly underrepresented relative to their industrial importance.
3. A translational taxonomy connecting biological principles, engineering abstractions, enabling technologies, and mechanical industrial use cases—with indicative TRL assessments—is proposed as a structuring tool for future research investment and technology readiness evaluation.
4. Five priority research gaps were identified and structured as observed pattern, engineering significance, and research opportunity: weak integration of locomotion and sensing payload; absence of field-deployment validation; underrepresentation of condition-monitoring and maintenance decision support; fragmentation across publication venues; and absence of standardised benchmarking.
5. The mapped evidence supports interpreting biomimetics in mechanical engineering as a functional framework for organising sensing, inspection, access, and maintenance-oriented research problems. This should be read as an interpretive synthesis derived from the structure of the literature rather than as a conclusively demonstrated property of the field. The field has generated substantial conceptual and prototypal activity, but the principal translational deficit remains the weak consolidation of biomimetic ideas into validated, benchmarked, and deployable industrial systems.

## Figures and Tables

**Table 1:** Inclusion and exclusion criteria applied in both screening stages.

Category	Criterion
Inclusion criteria	
Framing	Biomimetic, bioinspired, bio-inspired, bionic, or nature-inspired framing in title, abstract, or keywords.
Function	Relevance to at least one mechanical-engineering function: sensing, inspection, fault diagnosis, condition monitoring, locomotion, navigation, confined-space access, or adaptive control.
Evidence	Abstract sufficient to classify purpose, system type, and application context.
Type	Article, review, or conference paper with identifiable engineering contribution.
Exclusion criteria	
Domain	Dominant biomedical, clinical, tissue-engineering, drug-delivery, or chemistry content without translation to mechanical systems.
Specificity	No direct or foreseeable linkage to mechanical-system sensing, inspection, monitoring, robotics, or control.
Evidence	Abstract insufficient for functional topic assignment.
Duplication	Repeated title within the export.

**Table 2:** Functional clusters for topic assignment.

Cluster	Mechanical-Engineering Scope
Sensing and monitoring	Bioinspired sensors, tactile sensing, acoustic and vibration monitoring, structural health monitoring (SHM), fault diagnosis, predictive maintenance.
Vision and inspection	Machine vision, bioinspired imaging, defect detection, inspection workflows, non-destructive testing (NDT).
Robotics and access	Climbing, crawling, snake-like, aerial, and underwater platforms for confined-space access and inspection.
Control and computation	Adaptive control, bioinspired optimisation, neuromorphic computation, distributed decision logic for mechanical systems.
Mechanical surfaces and tribology	Adhesion, friction, wear, compliant mechanisms, aeroacoustic performance, and morphology-driven structural response.

**Table 3:** Screening pipeline summary and document-type distribution of the included set (n=505).

Screening Stage or Document Type	Records
*Screening pipeline*	
Scopus records retrieved	11,114
Duplicate titles removed	32
Records after deduplication	11,082
Stage 1: broad mechanical-industrial filter	570
Final included set after strict eligibility audit	505
*Document types in the included set*	
Article	323
Conference paper	103
Review	79

**Table 4:** Top source journals in the included set (n=505), synchronised with Figure 3.

Source Title	Records
*Journal of Bionic Engineering*	15
*Bioinspiration and Biomimetics*	14
*Biomimetics*	14
*Tribology International*	13
*IEEE Robotics and Automation Letters*	12
*Journal of Mechanisms and Robotics*	10
*Chemical Engineering Journal*	10
*Soft Robotics*	7
*Angewandte Chemie–International Edition*	7
*Advanced Functional Materials*	7
*Mocaxue Xuebao/Tribology*	6
*ACS Applied Materials and Interfaces*	6

**Table 5:** Functional cluster distribution of the included set (n=505).

Functional Cluster	Records	%
Robotics and access	235	46.5
Mechanical surfaces and tribology	141	27.9
Sensing and monitoring	106	21.0
Vision and inspection	14	2.8
Control and computation	9	1.8
**Total**	**505**	**100.0**

**Table 6:** Translational taxonomy for biomimetics in mechanical engineering. TRL = Technology Readiness Level (indicative).

Biological Principle	Engineering Abstraction	Enabling Technology	Industrial Use Case	TRL
Bat echolocation	Acoustic pulse–echo sensing	Ultrasonic transducers, DSP	Pipe NDE, wall-thickness measurement	5–7
Fish lateral line	Distributed hydrodynamic sensing	MEMS pressure arrays, flexible sensors	Subsea pipe monitoring, turbine intake	3–5
Insect compound eye	Wide-field, low-power machine vision	Multi-aperture optics, event cameras	UAV-based NDT, wide-area inspection	4–6
Gecko/insect adhesion	Reversible dry adhesion	Microstructured polymers, suction	Climbing robots for vessels, towers	4–6 [5,16]
Snake/earthworm locomotion	Undulatory/peristaltic propulsion	Modular actuators, compliant links	In-pipe inspection, duct access	4–6 [6,17]
Owl silent flight	Trailing-edge serration	Structured surfaces, additive mfg.	Wind-turbine blades, fan design	5–7
Octopus/cephalopod	Distributed compliance and grasping	Soft actuators, fluidic elastomers	Subsea maintenance robotics	3–5 [18]
Insect/mammal olfaction	Chemical gradient sensing	Electrochemical sensors, e-nose	Gas leak detection, plant monitoring	4–6
Tree frog wet adhesion	Wet adhesion on complex surfaces	Micropatterned elastomers	Inspection robots on wet/curved surfaces	3–5 [16]
Fish schooling	Multi-agent distributed coordination	Swarm algorithms, multi-robot comm.	Coordinated structural inspection	2–4

## Data Availability

The included record set is provided as Supplementary Table S1. Rule-based screening scripts are available from the corresponding author upon reasonable request. The source Scopus export is subject to Elsevier’s terms of use and cannot be redistributed.

## Supplementary Materials

The following supporting information can be downloaded at: https://www.mdpi.com/article/10.3390/biomimetics11050346/s1, Table S1: PRISMA 2020 checklist implementation for the present systematic mapping review.
