# Supplementary material for: Biomimetics as a Functional Engineering Framework for Mechanical Systems: A PRISMA-Guided Systematic Mapping of Sensing, Inspection, Access Robotics, and Condition Monitoring (2016–2026)
Source: Biomimetics (Basel). 2026 May 15;11(5):346. doi: 10.3390/biomimetics11050346 (PMC13204916; doi:10.3390/biomimetics11050346)
Supplement: Supplementary file 1 [file biomimetics-11-00346-s001.zip › biomimetics-4273568 PRISMA 2020 flow diagram.pdf]

Supplementary Figure S1. PRISMA 2020 flow diagram for the two-stage screening pipeline (Scopus, April 2026).

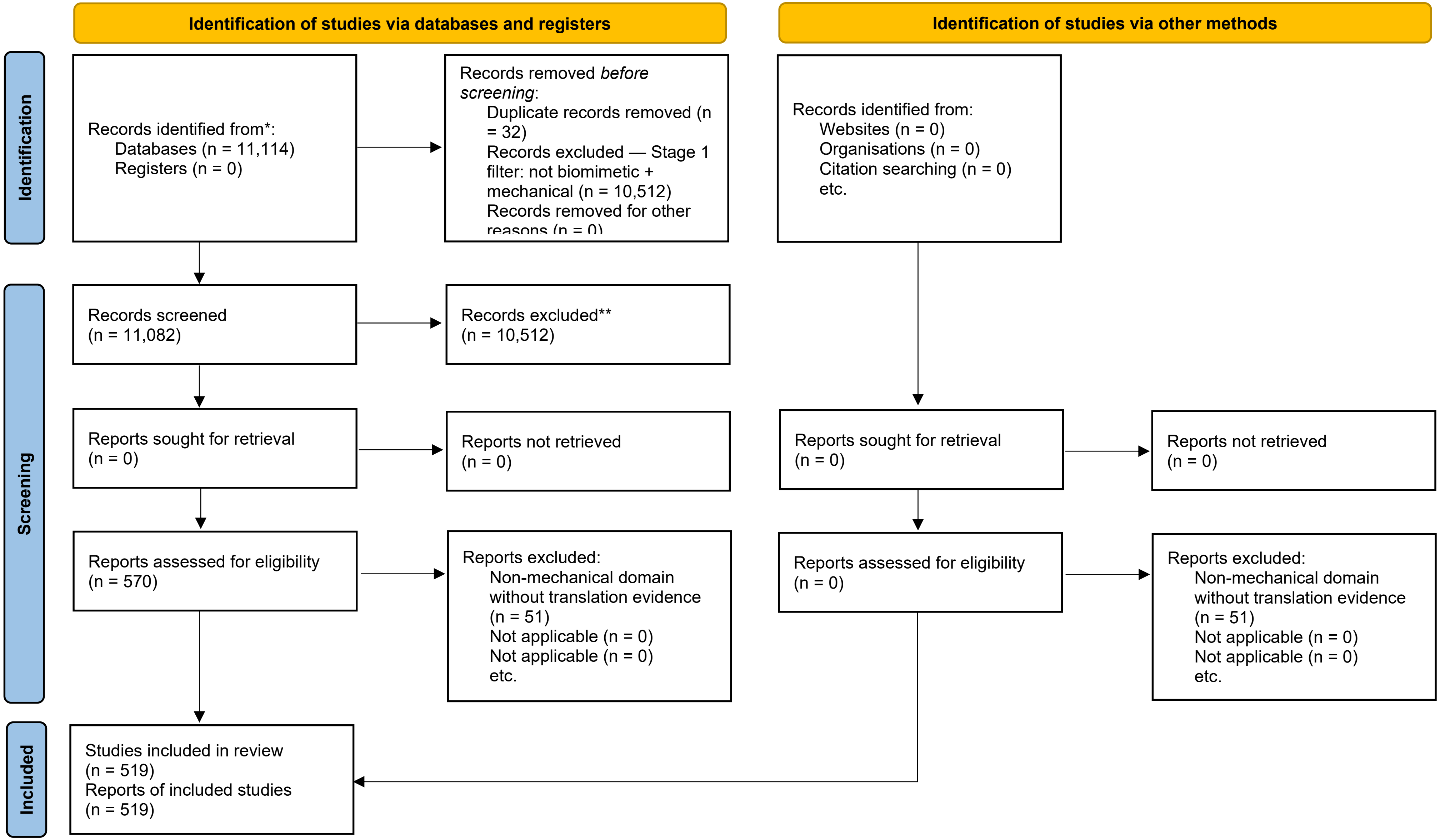

*Consider, if feasible to do so, reporting the number of records identified from each database or register searched (rather than the total number across all databases/registers).
**If automation tools were used, indicate how many records were excluded by a human and how many were excluded by automation tools.

Source: Page MJ, et al. BMJ 2021;372:n71. doi: 10.1136/bmj.n71.

Supplementary Figure S1. PRISMA 2020 flow diagram for the two-stage screening pipeline (Scopus, April 2026).

This work is licensed under CC BY 4.0. To view a copy of this license, visit https://creativecommons.org/licenses/by/4.0/
