# Supplementary material for: Biomimetics as a Functional Engineering Framework for Mechanical Systems: A PRISMA-Guided Systematic Mapping of Sensing, Inspection, Access Robotics, and Condition Monitoring (2016–2026)
Source: Biomimetics (Basel). 2026 May 15;11(5):346. doi: 10.3390/biomimetics11050346 (PMC13204916; doi:10.3390/biomimetics11050346)
Supplement: Supplementary file 1 [file biomimetics-11-00346-s001.zip › biomimetics-4273568 - supplementary.pdf]

Supplementary Materials: Supplementary Materials: Biomimetics as a Functional Engineering Framework for Mechanical Systems: A PRISMA-Guided Systematic Mapping of Sensing, Inspection, Access Robotics, and Condition Monitoring (2016–2026)

Cristóbal Galleguillos Ketterer [1,*], Nicolás Norambuena Ortega [1] and José Luis Valín [1]

Supplementary Table S1. PRISMA 2020 Implementation Map

The table below maps each PRISMA 2020 checklist item [1] against the corresponding location in the manuscript and indicates the implementation status for the current machine-assisted systematic mapping review. Items not applicable to the mapping review design are explicitly identified and justified.

Table S1. PRISMA 2020 checklist implementation for the present systematic mapping review.

Section	Item	Checklist requirement	Status	Location / note
Title	1	Identify the report as a systematic review.	✓ Met	Title specifies "PRISMA-Guided Systematic Mapping."
Abstract	2	Structured abstract: background, objectives, databases, eligibility, synthesis, results, conclusions.	✓ Met	Abstract section covers all required elements with quantitative results.
Intro	3	Rationale: describe the rationale for the review.	✓ Met	Section 1, paras. 1–4.
Intro	4	Objectives: explicit statement of review objectives.	✓ Met	Section 1, final paragraph; four objectives stated.
Methods	5	Eligibility criteria: specify inclusion and exclusion criteria.	✓ Met	Section 2.2, Table 1; defined *a priori*.
Methods	6	Information sources: specify databases, date of search.	✓ Met	Section 2.3: Scopus, April 2026.
Methods	7	Search strategy: full search string for each database.	✓ Met	Section 2.3: verbatim Scopus query provided.
Methods	8	Selection process: describe record screening and selection.	✓ Met	Section 2.4: two-stage machine-assisted process.

Table S1 – *continued*

**Section**	**Item**	**Checklist requirement**	**Status**	**Location / note**
Methods	9	Data collection: describe extraction method for each study.	✓ Met	Section 2.5: cluster assignment by keyword matching + adjudication.
Methods	10	Data items: list and define all variables extracted.	Partial	Functional clusters defined (Table 2); full variable list not itemised at record level. Noted as a limitation.
Methods	11	Study risk of bias assessment.	N/A	Mapping review; primary effectiveness assessment not performed. Explicitly noted in Section 4.5.
Methods	12	Effect measures: specify effect measures used.	N/A	No quantitative synthesis performed.
Methods	13	Synthesis methods: describe synthesis approach.	✓ Met	Section 2.6: descriptive and visual synthesis.
Methods	14	Reporting bias assessment.	N/A	Publication bias assessment not applicable to mapping reviews. Noted in Section 4.5.
Methods	15	Certainty assessment (GRADE or equivalent).	N/A	GRADE not applicable to mapping reviews.
Results	16a	Study selection: number of records at each stage with reasons.	✓ Met	Section 3.1; Figure 1 (PRISMA flow).
Results	16b	Reasons for exclusion at full-text stage.	N/A	No full-text review performed; deviation explicitly noted in Figure 1 caption and Section 4.5.
Results	17	Study characteristics: present characteristics of included studies.	✓ Met	Sections 3.2–3.5; Tables 2–4; Figures 2–5.
Results	18	Risk of bias in studies.	N/A	See item 11.
Results	19	Results of individual studies.	Partial	Supplementary Table S2 lists representative studies per cluster; individual study results not synthesised at record level.

Table S1 – *continued*

Section	Item	Checklist requirement	Status	Location / note
Results	20	Results of syntheses: cluster distribution, taxonomy.	✓ Met	Section 3.5 (Table 5); Section 3.6 (Table 6); Figures 6–8.
Results	21	Reporting biases across studies.	N/A	See item 14.
Results	22	Certainty of evidence.	N/A	See item 15.
Discussion	23	Discuss results, limitations, and implications.	✓ Met	Section 4: cluster analysis, gaps, connections to I4.0, limitations.
Other	24	Registration and protocol.	Not met	Review not prospectively registered. Explicitly noted as a limitation.
Other	25	Support: sources of funding.	✓ Met	Funding statement: no external funding.
Other	26	Competing interests.	✓ Met	Conflicts of Interest statement.
Other	27	Data, code, and material availability.	✓ Met	Data Availability Statement: screening file available from corresponding author on request.

Supplementary Table S2. Representative Studies by Functional Cluster

The following table lists representative included studies by functional cluster, selected to illustrate the range of topics and venues within each cluster. The list is not exhaustive; each entry was selected for high citation count, topic diversity, or direct relevance to the industrial use cases discussed in Section 4 of the main manuscript.

Table S2. Representative included studies by functional cluster. DOI links are active.

Cluster	Year	Title	Source
Robotics & access	2017	A crawling robot driven by multi-stable origami [2]	*Smart Mater. Struct.*
Robotics & access	2019	JumpRoACH: A trajectory-adjustable integrated jumping-crawling robot [3]	*IEEE/ASME Trans. Mechatron.*
Robotics & access	2020	Modeling of the in-pipe inspection robot: A comprehensive review [4]	*Ocean Eng.*
Robotics & access	2020	An 88-milligram insect-scale autonomous crawling robot driven by a catalytic artificial muscle [5]	*Sci. Robot.*
Robotics & access	2017	Adaptive control strategies for interlimb coordination in legged robots: A review [6]	*Front. Neurorobot.*

Table S2 – *continued*

Cluster	Year	Title	Source
Robotics & access	2016	Bioinspired dry adhesive materials and their application in robotics: A review [7]	*J. Bionic Eng.*
Robotics & access	2017	A survey on dielectric elastomer actuators for soft robots [8]	*Bioinspir. Biomim.*
Sensing & monitoring	2019	Bio-inspired intelligent structural color materials [9]	*Mater. Horiz.*
Sensing & monitoring	2021	Nature inspired emerging sensing technology: Recent progress and perspectives [10]	*Mater. Sci. Eng. R*
Sensing & monitoring	2022	Bioinspired sensor system for health care and human–machine interaction [11]	*EcoMat*
Sensing & monitoring	2016	Bio-inspired sensors based on photonic structures of Morpho butterfly wings [12]	*J. Mater. Chem. C*
Surfaces & tribology	2016	Biomimetic multifunctional surfaces inspired from animals [13]	*Adv. Colloid Interface Sci.*
Surfaces & tribology	2018	Tree frog attachment: Mechanisms, challenges, and perspectives [14]	*Front. Zool.*
Surfaces & tribology	2022	Tailored surface textures to increase friction—a review [15]	*Friction*
Vision & inspection	2013	Miniature curved artificial compound eyes [16]	*Proc. Natl. Acad. Sci. USA*
Control & computation	2024	Bioinspired sensing-memory-computing integrated vision systems: biomimetic mechanisms, designs, and applications [17]	*Sci. China Inf. Sci.*

Supplementary Table S3. Top Author Keywords in the Included Set

Table S3. Top 25 author keywords in the included set ($n = 519$), ranked by frequency.

Keyword	Occurrences
friction	20
biomimetic	20
bio-inspired design	15
legged robots	14
climbing robot	13
tribology	12
soft robotics	11
biomimetics	10
mechanism design	10
soft robots	10
bio-inspired	9

Table S3 – *continued*

**Keyword**	**Occurrences**
lubrication	9
bio-inspired robot	9
bio-inspired robotics	9
climbing robots	8
bioinspired	8
adhesion	8
wear resistance	7
biologically-inspired robots	7
fault diagnosis	6
structural health monitoring	5
condition monitoring	5
nondestructive testing	4
predictive maintenance	4
inspection robot	4

Note: The low frequency of fault diagnosis, structural health monitoring, condition monitoring, nondestructive testing, and predictive maintenance keywords relative to robotics and tribology terms quantitatively reflects the functional cluster imbalance discussed in Section 4.2 of the main manuscript and identified as priority research gaps in Section 4.3.

Supplementary Figure Notes

Figure 1 (PRISMA Flow)

The flow diagram follows the PRISMA 2020 template, adapted for a machine-assisted abstract-level screening workflow. The standard "full-text articles assessed for eligibility" node is replaced by the Stage 2 eligibility filter, with explicit notation that no full-text retrieval was performed. This deviation is an acknowledged methodological limitation.

Figure 2 (Temporal Evolution)

Three time series are plotted: full corpus (11,114 records), Stage 1 broad candidate set (570 records), and included set (519 records), 2016–2026. The post-2022 inflection is visible in all three series. The 2026 count is partial (search conducted April 2026).

Figure 3 (Document Types)

Article dominance (64.0%) is consistent with the applied, design-oriented character of the included literature. The elevated review proportion (16.2%) reflects maturing synthesis activity in bioinspired tribology, adhesion, and in-pipe inspection robotics.

Figure 4 (Top Source Journals)

The co-presence of biomimetics-specific journals (*Biomimetics*, *Bioinspiration & Biomimetics*, *Journal of Bionic Engineering*) and engineering-specialised venues (*IEEE Robotics and Automation Letters*, *Tribology International*, *Journal of Mechanisms and Robotics*) reflects the translational nature of the literature. The absence of major NDT or maintenance journals (*NDT & E International*, *Mechanical Systems and Signal Processing*) from the top-source list reinforces the observation that the biomimetics–maintenance interface is underserved.

Figure 5 (Term Frequency)

The dominance of locomotion, tribology, and adhesion vocabulary confirms the cluster-size distribution. Monitoring, diagnosis, and maintenance terms appear in the lower

frequency tier, providing a quantitative footprint of Research Gap 3 (underrepresentation of condition monitoring).

Figure 6 (Keyword Co-occurrence Network)

The network was generated from author and index keywords across the 519 included records. Edges represent co-occurrence in three or more records. The four visible clusters correspond approximately to the functional families. Low cross-cluster edge density between sensing and robotics keywords is the network-level signature of Research Gap 1 (integration of sensing payload with locomotion).

Figure 7 (Topic Distribution)

Robotics-and-access (45.3%) constitutes the plurality; vision-and-inspection (2.7%) and control-and-computation (1.7%) are the smallest clusters despite representing functions central to industrial inspection and autonomous maintenance.

Figure 8 (Functional Taxonomy)

The taxonomy figure illustrates the conceptual mapping from biological principles (left) through engineering abstractions and enabling technologies to industrial use cases (right). Colour coding corresponds to the five functional clusters in Table 2 of the main manuscript. TRL estimates are indicative (see Section 3.6).

Supplementary Note: PRISMA Flow Diagram—Textual Description

For editors or reviewers requiring a textual description:

Identification: Records from Scopus: **11,114**. Duplicate titles removed: **32**. Records after deduplication: **11,082**.

Stage 1 Screening (broad mechanical-industrial filter): Records screened: **11,082**. Records excluded (no mechanical-engineering function or biomimetic framing): **10,512**. Records retained: **570**.

Stage 2 Eligibility (strict mechanical filter): Records assessed: **570**. Records excluded (non-mechanical primary domain without compensating translation evidence): **51**. **Records included in final set: 519.**

Note: No full-text retrieval was performed. Both stages operated on title, abstract, and keyword fields from the Scopus CSV export. This constitutes a deviation from the standard PRISMA 2020 flow and is acknowledged as a methodological limitation.

1. Page, M.J.; McKenzie, J.E.; Bossuyt, P.M.; Boutron, I.; Hoffmann, T.C.; Mulrow, C.D.; Shamseer, L.; Tetzlaff, J.M.; Akl, E.A.; Brennan, S.E.; et al. The PRISMA 2020 statement: An updated guideline for reporting systematic reviews. *BMJ* **2021**, *372*, n71. 10.1136/bmj.n71
2. Pagano, A.; Yan, T.; Chien, B.; Wissa, A.; Tawfick, S. A crawling robot driven by multi-stable origami. *Smart Mater. Struct.* **2017**, *26*, 094007.
3. Jung, G.-P.; Casarez, C.S.; Jung, S.-P.; Fearing, R.S.; Cho, K.-J. JumpRoACH: A trajectory-adjustable integrated jumping-crawling robot. *IEEE/ASME Trans. Mechatron.* **2019**, *24*, 947–958.
4. Ab Rashid, M.Z.; Yakub, M.F.M.; Mahmed, N.; Shaari, M.F.; Bakar, E.A. Modeling of the in-pipe inspection robot: A comprehensive review. *Ocean Eng.* **2020**, *203*, 107206.
5. Yang, X.; Chang, L.; Pérez-Arancibia, N.O. An 88-milligram insect-scale autonomous crawling robot. *Sci. Robot.* **2020**, *5*, eaba0015.
6. Aoi, S.; Manoonpong, P.; Ambe, Y.; Matsuno, F.; Wörgötter, F. Adaptive control strategies for interlimb coordination in legged robots. *Front. Neurorobot.* **2017**, *11*, 39.
7. Li, Y.; Ahmed, A.; Sameoto, D.; Menon, C. Bioinspired dry adhesive materials and their application in robotics. *J. Bionic Eng.* **2016**, *13*, 181–199.

8. Gu, G.-Y.; Zhu, J.; Zhu, L.-M.; Zhu, X. A survey on dielectric elastomer actuators for soft robots. *Bioinspir. Biomim.* **2017**, *12*, 011003.
9. Shang, L.; Zhang, W.; Xu, B.; Zhao, Y. Bio-inspired intelligent structural color materials. *Mater. Horiz.* **2019**, *6*, 945–958.
10. Tripathy, A.; Pramanik, S.; Cho, J.; Santhosh, J.; Osman, N.A.A. Nature inspired emerging sensing technology. *Mater. Sci. Eng. R Rep.* **2021**, *144*, 100647.
11. Xue, J.; Chen, Z.; Wang, L.; Su, Y.; Li, X. Bioinspired sensor system for health care and human–machine interaction. *EcoMat* **2022**, *4*, e12209.
12. Li, Q.; Zeng, Q.; Shi, L.; Zhang, X.; Zhang, K.-Q. Bio-inspired sensors based on photonic structures of Morpho butterfly wings. *J. Mater. Chem. C* **2016**, *4*, 1752–1763.
13. Han, Z.; Fu, J.; Feng, X.; Niu, S.; Zhang, J.; Ren, L. Biomimetic multifunctional surfaces inspired from animals. *Adv. Colloid Interface Sci.* **2016**, *234*, 27–50.
14. Langowski, J.K.A.; Dodou, D.; Kamperman, M.; van Leeuwen, J.L. Tree frog attachment: Mechanisms, challenges, and perspectives. *Front. Zool.* **2018**, *15*, 32.
15. Costa, H.L.; Schille, J.; Rosenkranz, A. Tailored surface textures to increase friction—a review. *Friction* **2022**, *10*, 1255–1299.
16. Floreano, D.; Pericet-Camara, R.; Viollet, S.; Ruffier, F.; Brückner, A.; et al. Miniature curved artificial compound eyes. *Proc. Natl. Acad. Sci. USA* **2013**, *110*, 9267–9272.
17. Huang, Y.; Zheng, X.; Wang, Z.; Gao, Z.; Zhao, J.; Guo, T.; et al. Bioinspired sensing-memory-computing integrated vision systems. *Sci. China Inf. Sci.* **2024**, *67*, 121401.
